# Brominol

**Published:** 1901-06-08

**Authors:** 


### BROMINOL.

Notwithstanding the success which attends the use of bromide of potassium in many nervous diseases the necessity of administering day by day so large a quantity of alkali (for no other purpose according to some than the introduction of the accompanying bromine) has been regarded as an evil which it would be worth much to avoid. Various other vehicles have from time to time been suggested, and of late considerable use has been made of certain preparations in which the bromine is held in chemical union with some of the elements of certain essential oils. The question has however been raised whether these oily preparations are really decomposed in the intestine—whether, that is, the alkaline salts there met with are able to split up the comparatively stable combination of the bromine with the oil—and thus whether the bromine ever gets to the tissues as it does when given in the usual alkaline combination. At the suggestion of Sir William Gowers, Dr. Harrison Martindale, Ph.D., has lately made a very careful series of analyses of the urine and stools of patients taking roughly equivalent doses on the one hand of bromide of potassium and on the other of "brominol," an additive compound of bromine and sesame oil (the oil from the seeds of *Sesamum orientale*, one of the cotton-seed oil group). The results of the investigation go to show that this compound is thoroughly decomposed in the intestine, and that the bromine it contains gains access to the tissues, probably in the form of an alkaline bromide, and is ultimately discharged entirely by the urine. As to the therapeutic efficiency of such preparations it is perhaps too early to speak. It would seem, however, that one may properly expect to find that their mode of action is at any rate different from that of the alkaline salts. These are quickly absorbed and quickly excreted. The bromine from the oily preparation, however, is probably absorbed only gradually, no more quickly in fact than it is decomposed, and it is therefore reasonable to believe that its action will be equivalent rather to that which would be produced by the very frequent administration of small doses of bromides. All this, however, has yet to be worked out. As Sir William Gower says, "the clinical observations on its influence are not yet sufficiently numerous or decisive to justify their publication."

